# *N*-mixture models reliably estimate the abundance of small vertebrates

**DOI:** 10.1038/s41598-018-28432-8

**Published:** 2018-07-09

**Authors:** Gentile Francesco Ficetola, Benedetta Barzaghi, Andrea Melotto, Martina Muraro, Enrico Lunghi, Claudia Canedoli, Elia Lo Parrino, Veronica Nanni, Iolanda Silva-Rocha, Arianna Urso, Miguel Angel Carretero, Daniele Salvi, Stefano Scali, Giorgio Scarì, Roberta Pennati, Franco Andreone, Raoul Manenti

**Affiliations:** 1 Department of Environmental Science and Policy, University of Milan, Milano, Italy; 2 Laboratoire d’Ecologie Alpine (LECA), CNRS, Université Grenoble Alpes, Grenoble, France; 3 Universität Trier Fachbereich VI Raum-und Umweltwissenschaften Biogeographie, Universitätsring 15, 54286 Trier, Germany; 4 Museo di Storia Naturale dell’Università di Firenze, Sezione di Zoologia “La Specola”, Via Romana 17, 50125 Firenze, Italy; 5 Natural Oasis, Via di Galceti 141, 59100 Prato, Italy; 6 DISAT, Università degli Studi di Milano-Bicocca. Piazza della Scienza 1, 20126 Milano, Italy; 7 Department of Earth, Environmental and Life Science (DISTAV), University of Genoa, Genova, Italy; 8 CIBIO Research Centre in Biodiversity and Genetic Resources, InBIO, Campus de Vairão. 4485-661, Universidade do Porto, Vairão, Vila do Conde Portugal; 9 Department of Health, Life and Environmental Sciences, University of L’Aquila, 67100 Coppito, L’Aquila Italy; 10 Museo di Storia Naturale di Milano, Corso Venezia 55, I-20121 Milano, Italy; 11 Department of Biosciences, University of Milan, Milano, Italy; 12 Museo Regionale di Scienze Naturali, Via G. Giolitti, 36, I-10123 Torino, Italy

## Abstract

Accurate measures of species abundance are essential to identify conservation strategies. *N*-mixture models are increasingly used to estimate abundance on the basis of species counts. In this study we tested whether abundance estimates obtained using *N-*mixture models provide consistent results with more traditional approaches requiring capture (capture-mark recapture and removal sampling). We focused on endemic, threatened species of amphibians and reptiles in Italy, for which accurate abundance data are needed for conservation assessments: the Lanza’s Alpine salamander *Salamandra lanzai*, the Ambrosi’s cave salamander *Hydromantes ambrosii* and the Aeolian wall lizard *Podarcis raffonei*. In visual counts, detection probability was variable among species, ranging between 0.14 (Alpine salamanders) and 0.60 (cave salamanders). For all the species, abundance estimates obtained using *N*-mixture models showed limited differences with the ones obtained through capture-mark-recapture or removal sampling. The match was particularly accurate for cave salamanders in sites with limited abundance and for lizards, nevertheless non-incorporating heterogeneity of detection probability increased bias. *N-*mixture models provide reliable abundance estimates that are comparable with the ones of more traditional approaches, and offer additional advantages such as a smaller sampling effort and no need of manipulating individuals, which in turn reduces the risk of harming animals and spreading diseases.

## Introduction

Estimating species abundance is a pivotal task of species monitoring. Unfortunately, in most of cases detecting individuals of the target species can be challenging. Very often we are not able to detect all individuals present in a given place and time, and this may happen for several reasons, such as their elusive behaviour, cryptic habits or simply because of the limited ability of surveyors^1^. Therefore, the number of observed individuals rarely represents a reliable estimation of the number of individuals occurring in a given area.

Multiple approaches have been developed to estimate the true number of present individuals. Among them, approaches requiring multiple sessions of capture have a considerable success. For instance, in capture-mark-recapture (CMR) approaches animals of a population are captured, individually marked or photographed to allow identification, and released at the capture site. The frequency of marked individuals observed in subsequent capture sessions is then used to estimate abundance^1,2^. Removal sampling (sometimes named catch-effort model) is an alternative approach, which requires the systematic capture and removal of individuals. Population size is then estimated on the basis of the decline in catch size during sequential capture sessions^2–4^. These approaches have been particularly useful to obtain reliable estimates of population size, and have revealed long-term temporal trends, allowing to identify the factors determining the survival of individuals and the decline of populations^5–9^.

However, approaches requiring the capture and identification are generally labour intensive, as many individuals need to be captured and identified to obtain reliable population estimates. Therefore, the broad scale monitoring of the abundance of wildlife has been a challenge for decades^10^. In the last years formal approaches have been proposed to estimate animal abundance from repeated counts at fixed sites, without marking individuals to identify them^11,12^. The number of individuals detected in a given site is counted using standard monitoring techniques (e.g., point counts, observation in small plots, fixed area transects), and each site is generally surveyed in multiple occasions. The repeated counts in a given site are then used to jointly estimate the detectability of individuals and population size based on *N-*mixture models^11–14^. As they do not require capture or manipulation of individuals, such models might allow collecting abundance information over larger areas compared to traditional approaches, can be appropriate for protected species, and have been proposed for broad-scale assessment of populations^13,15,16^. The usefulness of *N*-mixture models to estimate abundance of amphibians and reptiles is advocated since several years^1,13,17^ and, given their high cost-effectiveness, these approaches have also been suggested to obtain broad scale estimates of the population trends of amphibians and reptiles^16^. For instance, repeated counts analysed with *N*-mixture models have been proposed for the periodic monitoring of several species of amphibians and reptiles listed in the EU Habitat Directive at the national scale^18^.

Nevertheless, approaches based on *N*-mixture models are not yet widely used to estimate population abundance, perhaps because practitioners remain unsure about their efficiency compared to more traditional techniques requiring capture. Recent analyses casted doubts about the usefulness of *N*-mixture models, because the loss of information resulting from not marking animals can make problematic the joint estimation of abundance and detection probability^19^. Moreover, these models are sensitive to violations of their assumptions, and unmodeled heterogeneity in abundance or detection probability can cause substantial biases^20,21^. Thus, real-world studies are required to verify the estimates from *N*-mixture models under a range of conditions. Recently, Kéry^22^ found excellent agreement between estimates under *N*-mixture and those from different approaches (i.e., multinomial *N*-mixture models), which are a generalization of CMR that do not suffer from borderline estimability of parameters^19^. Furthermore, some studies comparing the performance of mixture models with more traditional approaches (e.g., CMR) found similar abundance and density estimates [e.g.^23,24^] but, until now, such comparative analyses have focused on a limited range of species.

In this study, we compared population estimates obtained using *N*-mixture models with estimates obtained applying more traditional approaches, i.e., removal sampling and capture-mark-recapture. We focused on three threatened species of amphibians and reptiles endemic of Italy and adjacent areas: the Lanza’s Alpine salamander *Salamandra lanzai*, the Ambrosi’s cave salamander *Hydromantes ambrosii* and the Aeolian wall lizard *Podarcis raffonei*. All these species are threatened, and both salamanders are fully terrestrial and do not require water for reproduction^25^, thus other traditional approaches to estimate the abundance of amphibians (e.g., egg counts, monitoring of breeding sites) cannot be used. Therefore, the reliability of monitoring approaches based on the observation of unmarked active individuals is a key aspect to provide effective information for management plans.

## Results

### Lanza’s Alpine salamander

**Figure 1:**
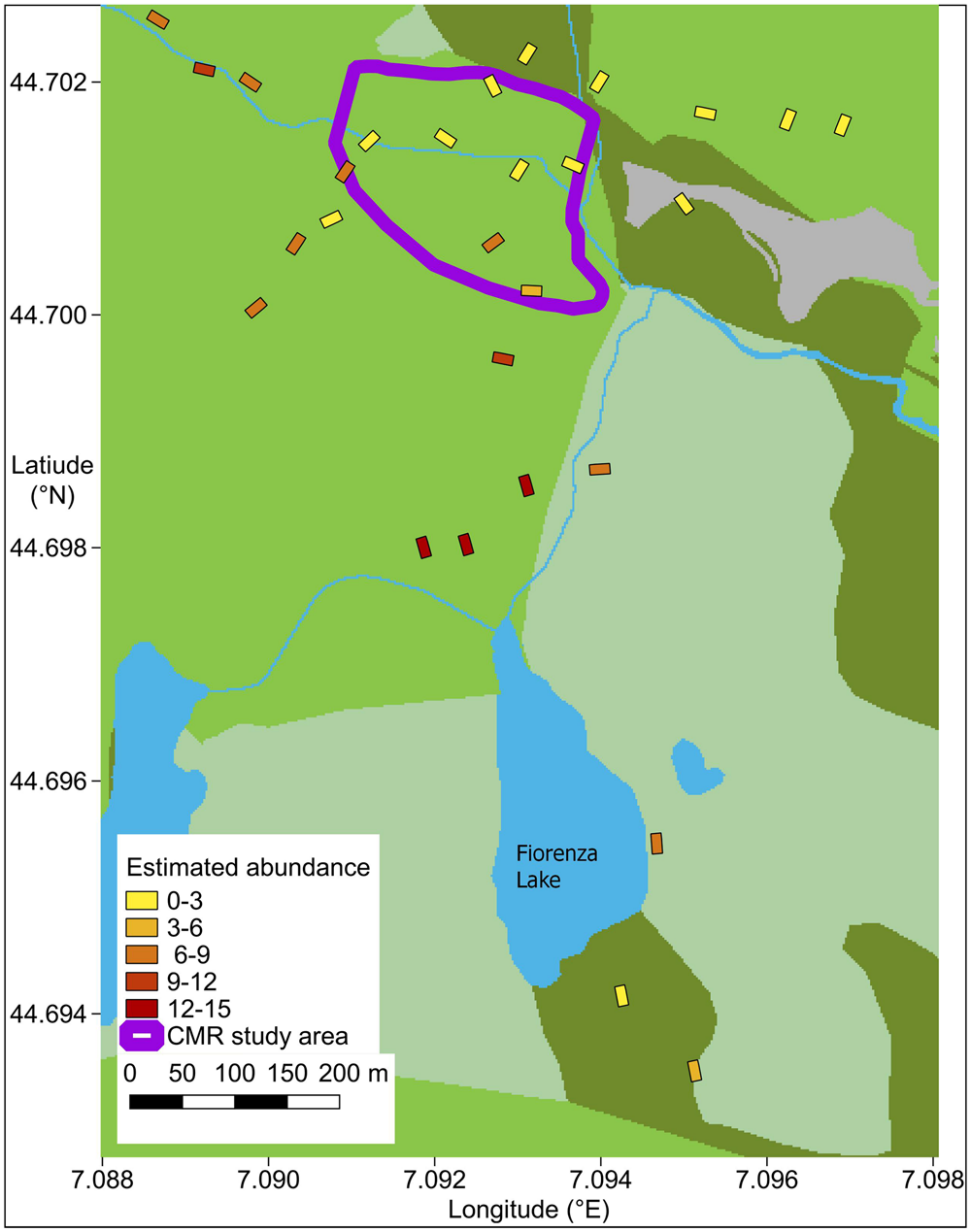
Plots used to assess the abundance of *Salamandra lanzai*, and spatial variation of abundance estimates. The violet line is the approximate limit of the area sampled with capture-mark-recapture^42^. The map was generated by GFF using the open-source software QGis 2.18 (QGIS Development Team, 2016. QGIS Geographic Information System. Open Source Geospatial Foundation Project. www.qgis.org); background colors represent land use (grey: built-up; green: pasture; pale green: sparse vegetation; dark green: high-altitude pasture and moorland; blue: water).

During 63 repeated surveys, we obtained 63 salamander detections (range: 0–9 individuals per plot in each survey). In *N-*mixture models, we used a zero-inflated Poisson model as it showed AIC values lower than the Poisson model (model without covariates: AIC: 180.9 vs. 207.2). *N-*mixture models estimated an average detection probability of 0.14 (95% CI: 0.02–0.62). The model including hour of survey as covariate showed a higher AIC value than the model without hour (AIC = 182.5), and hour did not have a significant effect on salamander detection (*z* = −0.20, *P* = 0.608), therefore we kept the model without covariates. The estimated number of individuals ranged between 0.4 and 14.7 individuals per plot (Fig. 1).

Studies using capture-mark-recapture^26,27^ estimated a density of ~300 individuals/ha. If we only consider the six plots within the CMR study area, the average density of salamanders estimated using *N*-mixture models was 141 individuals/ha (95% CI: 38–450 individuals/ha). However, if we also include the plots nearby the CMR study area, the average density of salamanders was higher, and closer to the estimates obtained using CMR. For instance, if we also consider plots within 250 m, the average density was 254.3 individuals/ha (95% CI: 130–544).

### Ambrosi’s cave salamander

During 20 repeated counts, we obtained 599 salamander detections (range: 0–123 detections per cave in each survey). In *N-*mixture models, we used a Poisson error distribution as it showed AIC values lower than zero-inflated Poisson models (AIC: 510.3 vs. 512.4). *N-*mixture models estimated an average detection probability of 0.62 (95% CI: 0.59–0.76); empirical Bayes estimates indicated population abundances between 13 and 135 individuals/site (Table 1).

**Table 1:** Abundance estimates in ten populations of *Hydromantes ambrosii*, obtained with different approaches.

Cave	*N* max	*N*-mixture models		Removal sampling		
		Abundance	95% CI	Capture rate	Abundance	95% CI
Pignone left entrance	27	33.8	29/39	0.5	24	*
Pignone right entrance	38	50.1	45/56	0.702	53	51/63
Pignone main cave	38	53.3	48/59	0.636	59	57/70
Pignone – False snake’s hole	5	11.0	7/16	†	†	
Pignone – Ambrosi’s sinkhole	3	8.7	5/13	†	†	
Fornace	30	43.3	38/49	0.38	76	*
Fornace left entrance	15	23.8	19/29	0.6	20	19/33
Pignone abandoned mine	52	57.8	54/63	0.386	114	92/240
Spelerpes	6	13.1	9/18	0.426	13	*
Alta di Castè	123	144.5	138/152	0.382	244	219/300

*N* max: max number of individuals detected in one single survey session.

^†^The method was unable to estimate population size.

^*^Estimation of 95% CI was not available.

During the removal experiment (*N* = 29 capture sessions), we captured 507 individuals (range: 0–99 individuals per cave in each session). In removal models, estimates of population size ranged between 13 and 244 individuals per cave (Table 1). The depletion method was unable to estimate population size for the two cavities with less captured individuals (False Snake and Ambrosi’s sinkhole), in which zero individuals were captured during the second and third capture sessions.

**Figure 2:**
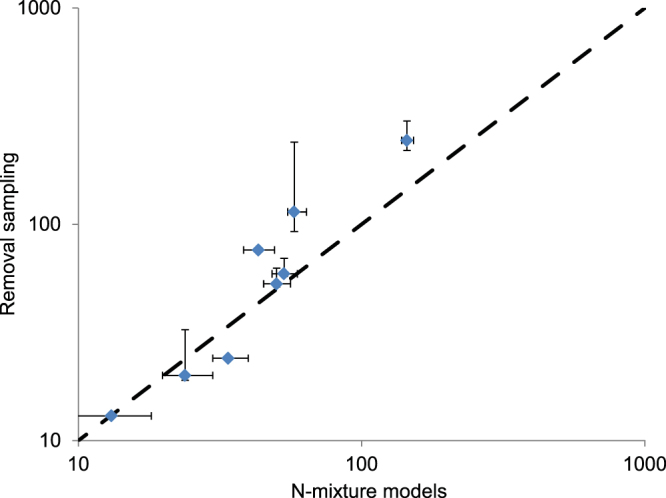
Abundance of *H. ambrosii*: comparison between removal sampling and *N*-mixture models. Error bars are 95% confidence intervals of each estimate, the black dashed line has intercept = zero and slope = 1.

Overall, *N-*mixture models and removal sampling provided highly correlated and consistent estimates of population densities (Fig. 1), with similar values in most of populations (Table 1, Fig. 2). In the populations with more individuals, *N*-mixture models tended to estimate smaller population sizes (Table 1). Nevertheless, a linear regression model, relating log-transformed abundances estimated with the two approaches revealed a strongly significant relationship (*R*^2^ = 0.91; *F*_1,6_ = 57.2, *P* < 0.001), with a slope not significantly different from one (*B* = 1.32, 95% CI = 0.89/1.74) and an intercept not significantly different from zero (*k* = −1.00, 95% CI = −2.63/0.61).

### Aeolian lizard

During 11 repeated counts, we obtained 85 detections of adult lizards in four replicated transects. On average, we observed 11.8 lizards/man-hour of survey. In *N-*mixture models, we selected the model with Poisson error, as it showed AIC values lower than the respective ZIP model (69.4 vs. 71.4). The model including hour of survey as covariate showed the lowest AIC value, and suggested that detection probability was highest in early morning (Table 2). At 9.30 a.m., the average detection probability was 0.18 (SE = 0.78). *N*-mixture models yielded a population size estimate of 274 individuals within the transects (95% CI: 221–334), indicating an average lizard density of 0.35 individuals/m^2^ (95% CI: 0.28–0.43). Assuming homogeneous density in the survey area, this yielded a total estimate of 1050 individuals (95% CI: 847–1280).

**Table 2:** Candidate *N*-mixture models on factors determining the detection probability of Aeolian lizards.

Variables in the model	K	AIC
hour of survey (−)	3.00	53.5
hour of survey (−); sampling effort (+)	4.00	55.1
None	2.00	69.4
sampling effort (+)	3.00	71.1

Signs after variable names indicate the sign of regression coefficients. Models are ranked on the basis of their AIC values. K: number of parameters in the model.

During the removal sessions (*N* = 5 capture sessions), we captured 131 adult lizards (63 males and 68 females; range: 7–18 males per capture session). Average capture rate was 2.9 individuals/man-hour. Males were temporarily maintained in terraria for the removal experiment, while most of females were immediately released in nature. The removal method estimated a total population size of 538 males (lambda: 0.003). If we assume a 1:1 sex ratio, the overall abundance of *P. raffonei* estimated using removal sampling was ~1080 individuals. The number of captured males per hour only slightly decreased from the first to the last capture session, therefore confidence intervals for this estimate were not available. Genetic analyses are currently ongoing to ascertain whether these lizards are pure *P. raffonei*, or are hybridized with non-native species.

## Discussion

Accurate estimates of population size provide baseline data for many studies on population ecology, are essential to assess the conservation status of populations, and allow to identify management priorities. For instance, in the European Union the Habitat Directive protects several hundreds of animal species, and requires regular reports on the conservation status of all species protected by the directive. Such reports should include measures of population size and trends for all these species across the continent. Obtaining quantitative measures of population size of hundreds species over broad areas requires considerable monitoring efforts, and volunteers are a key resource for such a broad scale monitoring^13,15,28–31^. Approaches based on the capture of individuals such as capture-mark-recapture or removal sampling can provide reliable estimates of population size, but also have drawbacks. First, the capture of many individuals often requires more time than just observing their presence, therefore it can be less effective if we need monitoring many populations. For instance, for lizards the detection rate was four times higher than the capture rate. Similarly, for cave salamanders the removal sampling required approx. 80 man-days of work, while only 28 man-days were required for the visual surveys of *N-*mixture models. Second, some techniques used to mark amphibians and reptiles are expensive, or can harm individuals and pose ethical issues (e.g., toe clipping)^32^. Finally, the manipulation of specimens can pose threats to the study populations, such as the risk of transmission of infectious diseases^33^. Actually, European salamanders currently face the risk of infection by the chytrid fungus, *Batrachochytrium salamandrivorans*, which is lethal to most salamanders and is causing dramatic declines in several populations^34–36^. Under these circumstances, protocols requiring the capture of individuals by a large number of volunteers cannot be advocated. *N*-mixture models just require the observation of individuals, and thus are a promising alternative that does not require the manipulation of individuals. Our study shows that this approach can provide reliable estimates of population size, which are highly comparable with the ones obtained by more traditional approaches.

Simulations suggest that population sizes estimated through *N*-mixture models generally have a limited bias if model assumptions are met^16,20^, but the accuracy of these analyses still requires assessment. If detection probability is ~0.15 (as we recorded for the Lanza’s salamander) and sites are surveyed only a few times, simulations suggested that the relative bias of mixture models may be 45–50%^16^, i.e., they can over- or underestimate population size by approx. 50%. The accuracy of mixture models increases in easily detectable species and, if detection probability is 0.5, the expected bias is ~12–20%, and the correlation between true and estimated population size is expected to be ~0.9 [Fig. S2 in^16^]. Testing the validity of these predictions is difficult, as in real populations we hardly know the true population size. Nevertheless, if we compare mixture models with CMR and removal sampling, we obtain measures of bias that are in agreement with these predictions. For Lanza’s Alpine salamanders, the differences between CMR and mixture models was 17–52% (depending if we consider all transects nearby the CMR study area, or only transects within the CMR study area; see below). For cave salamanders, the average relative bias was 26%, and the correlation between the two population size estimates was 0.95. The match between empirical data and simulations confirms the robustness of conclusions obtained through the virtual ecologist approach^37^, and supports mixture models as a reliable tool for the analysis of monitoring data.

Nevertheless, the reliability of *N-*mixture models heavily depends on meeting model assumptions, and recent simulations suggested that emigration, double counts or variation of detection probability among sampling occasions can produce biased results, yielding substantial overestimates of species abundances^20,21^. In our study, detection probability of lizards was not constant, and declined in surveys performed during late morning, as commonly observed for reptiles^38^; actually, the hour of survey was included in the best-AIC model (Table 2). It should be remarked that abundance estimates would be heavily biased, if such variation of detection probability was not incorporated into models. In fact, a model without detection covariates (e.g., model 3 in Table 2) would provide density estimates of 1.3 individuals/m^2^, which are three-times larger than the ones obtained through the removal experiment. The correct incorporation of heterogeneity of detection probability is thus essential to obtain useful estimates^20^, and appropriate knowledge of species biology can be extremely important to identify and accurately measure the variables that can allow describing this variability.

In the Lanza’s Alpine salamander example, the two population estimates (*N*-mixture vs. CMR) were not performed in the same year. It is well known that salamander populations can undergo strong temporal variation, for instance in response to habitat modifications, climatic variation and variation of biotic factors (e.g.^7,39,40^), and population fluctuations can occur even in absence of evident habitat changes^41^. Despite we do not have quantitative data on population dynamics, the available information suggests that the study populations did not undergo strong variations of abundance through time. For instance, for Alpine salamanders, CMR estimates of abundance obtained in 1992 and in 2003 were very similar^27,42^. Furthermore, the study area is a protected site, for which no major habitat modifications occurred in the last 20 years. Differences between CMR and *N*-mixture models were stronger if we only consider the eight plots falling within the CMR area. However, only eight plots were inside the target area, and sampled just 1,600 m^2^, which represent 3.9% of the surface sampled by CMR. Therefore, the imperfect match between the estimates possibly occurred because the sampling effort inside the target area was too low. Conversely, if all the plots nearby the Andreone *et al*.^25,27^ study area are considered, *N*-mixture models sampled a much larger surface (5,400 m^2^). Salamander distribution is not homogeneously distributed across the landscape (Fig. 1), and the more intense effort probably allows a better representation of the overall landscape. The quality of population estimates generally increases at high sampling efforts^43^ which, in this case, is related to both the number of surveys per plot, and the total area covered by plots. When planning surveys, both these parameters must be defined a priori. Increasing the surface of each plot, and increasing the number of plots, are alternative approaches to increase sampling efforts. Deciding the best strategy (a few large or several small plots) strongly depends on parameters such as population density, detection probability, spatial heterogeneity and logistic constraints, and should be decided a priori, on the basis of study aims and resources availability. For instance, the number of individuals that are detected at each survey is generally higher in larger plots. Therefore, large plots and/or a large number of surveys per plot are a more effective strategy for species with limited detectability, while surveying several small transects can be preferable if populations have high detection probability^16,44–46^.

With cave salamanders, detection probability estimates were very high (≥0.4) using both approaches. High detection probability has already been demonstrated in other species of cave salamanders, particularly during their underground activity phase^5,47–49^, and this favors studies on the ecology and dynamics of cave salamander populations (e.g.^39,48,49^). The match between mixture models and removal sampling was excellent for the caves with fewer salamanders. Mixture models tended to underestimate population size in the two caves where removal estimated more individuals (Table 1, Fig. 2). It should be remarked that capture rate, estimated by removal sampling, is unrelated to both cave depth and salamander abundance (|r| ≤ 0.4 and *P* > 0.25 for both correlation), suggesting that this does not occur because sampling quality was weaker in larger caves and/or in caves with more salamanders. Overall, the slope of the regression between population sizes estimated with the two approaches was not significantly different from one, and in the majority of cases abundance estimates were extremely similar confirming that, in most of sites, *N*-mixture models are an excellent approach to estimate the abundance of these animals. Nevertheless, additional analyses are needed to understand the performance of *N*-mixture models when variation of abundance among sites is strong.

We showed that *N*-mixture models can provide effective measures of the abundance of populations for small vertebrates with very different habits and living in a wide range of habitats, from nocturnal salamanders living in alpine meadows, to lizards living in Mediterranean islands. However, just measuring abundance provides limited information for conservation. An additional advantage of *N*-mixture models is the possibility of including covariates as potential predictors of species abundance also at very fine spatial scale^17^. Assessing the factors that can determine differences in abundances among sites, or differences in abundance in a site surveyed during different years would provide more complete information and, for instance, can allow the identification of threatening factors that should be targeted by conservation strategies^50,51^. During surveys, experienced observers can also record parameters representing habitat quality or threats^51^ that can be successfully integrated within *N*-mixture models to provide quantitative management indications (e.g.^17^). The elaboration of comprehensive monitoring schemes, that allow the integration of species abundance data with information on habitat features and on drivers of abundance is not easy^52^, but will provide essential information for more effective management.

## Methods

### Species, study areas and sampling

#### Lanza’s Alpine Salamander Salamandra lanzai

*Salamandra lanzai* is endemic of a small area of the Alps, between NW Italy and E France, and lives at altitudes of 1200–2650 m. This salamander is viviparous, and individuals are active at the surface from late spring until early autumn, mostly at night and during humid periods^26^. The study was performed at the Pian del Re area (approx. 44.70°N, 7.10°E; altitude: 2000–2150 m; Fig. 1), which is an alpine meadow with multiple small streams, and is the type locality of *S. lanzai*.

*Capture-mark-recapture* data were obtained from previously published studies performed in 1992–2003, which sampled a surface of approx. 41,000 m^2^ (Fig. 1 ^27,42^). *Repeated counts*. We defined 28 rectangular (20 × 10 m) plots, across the whole Pian del Re. Each plot was surveyed by a 6–8 people team, actively searching and counting all the salamanders at the surface for 10–15 min. Plots were surveyed 2–3 times (average: 2.3 surveys per plot) in the period 16–19 August 2015 after dusk, between 9.00 pm and 1.00 am. We positioned the 28 plots performed in 2015 as follows: eight were inside the study area where Andreone *et al*. (refs^27,42^) performed their CMR study, 17 were nearby the Andreone *et al*. (refs^27,42^) study area (<250 m from the area), and three were 500–750 apart (Fig. 1).

#### Cave salamander Hydromantes ambrosii

The Ambrosi’s cave salamander *H. ambrosii* (see^53^ for discussion on nomenclature) is endemic of a small area of peninsular Italy. This terrestrial salamander does not live exclusively in caves, as from autumn to spring it is often active at the surface. However, during the dry and hot Mediterranean summer it often moves to underground refugia, where its detectability is highest^48,54^. In June 2017, we monitored ten cavities (caves and abandoned mines) in Central Italy using both repeated counts and removal sampling. We considered the Spelerpes cave (44.13°N, 9.78°E), six cavities within the Pignone karst Area (44.18°N, 9.72°E) and the Alta di Castè cave (44.12°N, 9.77°E). Explored depth of caves ranged between 9 and 48 m. For repeated counts, each cave was monitored by 6–7 observers during daytime in sunny, dry days, by actively searching and counting all the salamanders visible on both cave walls and floor, as described by Lunghi, *et al*.^48^. Each cave was surveyed two times within 3–10 days, to ensure meeting assumption of population closure. Survey effort was approx. 0.5 man/hour for each m of explored cave. Subsequently, we performed a three-sample removal experiment^4^. Immediately after the end of the last count survey, 6–7 people collected and stored salamanders in specific fauna boxes (40 × 25 × 15 cm) which were left inside caves. Removal session ended after 10 minutes without captures. At the end of the third session of capture, animals were released in the same cave areas in which they were found. Individuals were manipulated with disposable nitrile gloves, and all the equipment was disinfected following guidelines for preventing the spread of infectious diseases^55^.

#### Aeolian wall lizard Podarcis raffonei

The Aeolian wall lizard *Podarcis raffonei* is endemic of the Aeolian Archipelago (Southern Italy). The species is critically endangered and is undergoing a quick decline; the most likely factors determining lizard decline are the competition/hybridisation with non-native lizards, and habitat modifications. Currently, only four populations of this species are known to persist^56,57^. During spring 2017 (end of April-beginning of May) we carried out field surveys in the Capo Grosso Peninsula (approx. 38°25′N, 14°56′E; surface area available for surveys: 2990 m^2^) in order to estimate the size and the status of the last population of *P. raffonei* on the Vulcano Island using repeated counts (visual strip transects) and removal sampling. First, we established four linear transects (length: 60–75 m; width: 1.5 m) covering the whole peninsula, and used visual encounter surveys to count the number of active lizards. Transects were >5 m apart, to avoid double counts of the same individual, and were performed between 9.00 and 12.00 a.m. by one-two observers. Only adult lizards were considered; each transect was repeated 2–3 times (average: 2.75). Second, individuals were noose-captured through the whole peninsula. Females were mostly released immediately after capture, while all the captured males were temporarily transported in terraria. Overall, we performed five capture sessions; the sampling efforts of capture sessions ranged between six and 12.5 man/hours.

### Data analysis

For all the species, repeated counts were analysed using *N*-mixture models for closed populations^11^. We used Akaike’s Information Criterion^58^ to select the most appropriate error distribution (Poisson or zero-inflated Poisson); we did not consider negative binomial errors as can produce infinite abundance estimates, particularly when detection probability is limited^59^. In models, we used 100+ the maximum observed species abundance as upper bound to approximate an infinite summation of the likelihood, since preliminary analyses suggested that this value provides robust estimates^16^. For Lanza’s Alpine salamanders, activity is often higher early after dusk^42^, thus we considered hour of survey as a covariate potentially affecting detection probability; all surveys were conducted within four days, with constant meteorological conditions (similar temperature; no rain). For Aeolian lizards, all surveys were performed within one week, during sunny days without wind. However, activity of lizards is generally higher in early morning, and survey effort was variable (range: 0.33–0.66 man/hours per transect), therefore we tested models including hour of survey and survey effort as detection covariates. For all the species we then used empirical Bayes methods to estimate the posterior distribution of the abundance (mean and 95% Bayesian credible intervals)^60^. *N-*mixture models were run using the unmarked package in R 3.3.3^61^.

To estimate population size from removal sampling of *H. ambrosii* and *P. raffonei*, we used the sampling coverage estimator for heterogeneous models of Chao and Chang^4^, which is able to estimate population size with low bias, assuming that capture rate can be different among individuals. For *P. raffonei*, the length of sampling session (men-hours) was included as a measure of sampling effort. In preliminary analyses, we also tried using methods assuming homogeneous detection probabilities^4^, and obtained very similar estimates.

### Availability of data and materials

The dataset supporting the conclusions of this article is included within the additional files.

### Ethics statement

All research involving animals was performed in accordance with the national regulations, and was conducted under the authorization of National Authorities (Ministero dell’Ambiente della Natura e del Mare; *H. ambrosii*: 9384/PNM/2015, 20624/PNM/2016; *S. lanzai*: 14382/PNM/2015, 12273/PNM/2015; *P. raffonei*: 4602/PNM/2017).

## Electronic supplementary material

Appendix 1
